# Supplementary material for: On-chip phonon-enhanced IR near-field detection of molecular vibrations
Source: Nat Commun. 2024 Oct 16;15:8907. doi: 10.1038/s41467-024-53182-9 (PMC11484778; doi:10.1038/s41467-024-53182-9)
Supplement: Supplementary file 1 — Supplementary Information [file 41467_2024_53182_MOESM1_ESM.pdf]

Supplementary information for

"On-chip phonon-enhanced IR near-field detection of molecular vibrations"

A.Bylinkin et.al.

Table of Contents

Suppl. Note 1. Additional information about the fabrication of the graphene split-gate detector.. 2

Suppl. Note 2. Electrical characterization of the detector 3

Suppl. Note 3. Schematic of the Fourier transform infrared spectroscopy setup 4

Suppl. Note 4. Optical characterization of the graphene split-gate detector 5

Suppl. Note 5. Calculation of optical absorption in graphene 8

Suppl. Note. 5.1. Electrostatic simulations 8

Suppl. Note. 5.2. Dielectric functions of materials used in electromagnetic simulations 8

Suppl. Note. 5.3. Electromagnetic simulations 10

Suppl. Note. 5.4. Influence of incident light polarization on absorption in graphene 11

Suppl. Note 6. Influence of gap size, h-BN thickness and HPhPs on graphene absorption 12

Suppl. Note 7. Influence of CBP background permittivity on normalized absorption in graphene in the molecule-covered detector 17

Suppl. Note 8. Convolution of the normalized absorption and transmission spectra 18

Suppl. Note 9. Data set of the experimental on-chip SEIRA and far-field transmission spectra . 20

Supplementary references 21

Suppl. Note 1. Additional information about the fabrication of the graphene split-gate detector

Suppl. Fig. 1a shows a schematic top view of the graphene split-gate detector. The indicated dimensions were determined from the scanning electron microscopy (SEM) image shown in Suppl. Fig. 1b. The dimensions of the graphene channel, source and drain contacts were determined from the atomic force microscopy (AFM) image shown in Suppl. Fig. 1d. An optical image of the fabricated device is shown in Suppl. Fig. 1c.

Suppl. Fig. 1d shows the AFM image of the detector surface after brooming[1] (see Methods), demonstrating that the centre part of the detector is clean and free from lithographic residues, ensuring that the molecular layer was evaporated directly onto the surface of the top h-BN layer of the detector.

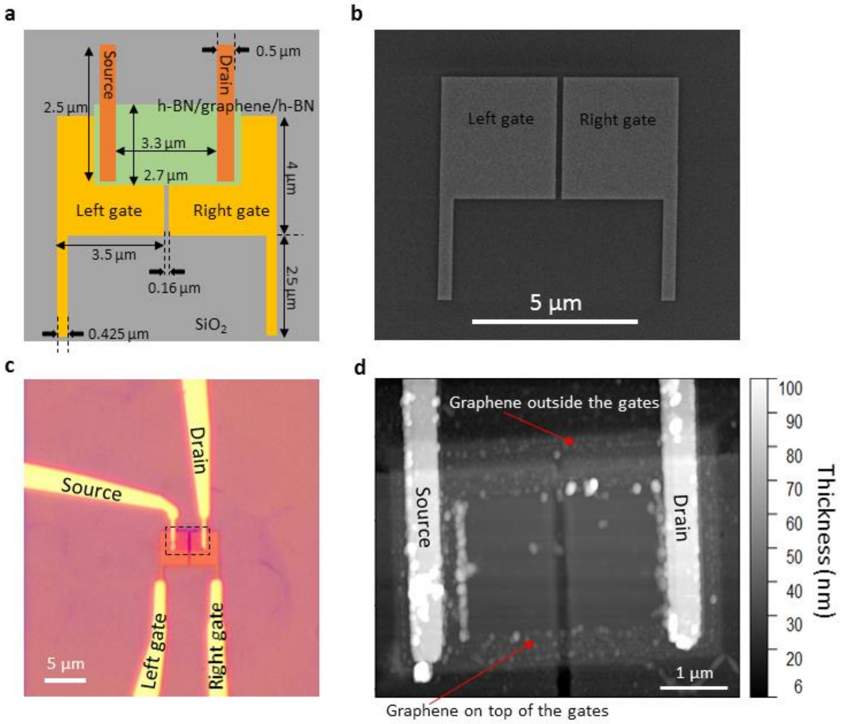

Supplementary Figure 1. Graphene split-gate detector. **a,** Schematic of the detector with the dimensions of the split-gate, graphene channel, source and drain electrodes obtained from the SEM image shown in panel **b**. The dimensions of the graphene channel, source and drain contacts were determined from the AFM image shown in panel **d**. **b**, SEM image of the fabricated bare split-gate. **c,** Optical image of the fully fabricated detector. The dashed square marks the area shown in panel **d**. **d,** AFM image of the graphene split-gate detector after brooming. Red arrows indicate areas of graphene that are located outside the split-gate.

In Suppl. Fig. 1d we also observe that the graphene is partially located outside the split-gate. This finding may explain the different charge neutrality points of graphene obtained from the electrical and optical measurements (Suppl. Note 2, 4).

Suppl. Note 2. Electrical characterization of the detector

We applied 50 mV bias between the source and drain contacts and measured the current, from which we obtained the resistance, R. Suppl. Fig. 2 shows the resistance as a function of the gate voltage, which was applied to both gates simultaneously. Using the electrical circuit model from references [2,3], we fitted the resistance data. Electron and hole mobilities in graphene, $\mu_{e,h}$, residual local charge fluctuations, n_0^*, and contact resistance were fit parameter. We obtained $n_0^* = 5\times10^{11}$ cm^{-2}, an average contact resistance of 1150 Ω (2.6 kΩ μm), and electron and hole mobilities of 11200 $cm^2\,V^{-1}\,s^{-1}$ and 8100 $cm^2\,V^{-1}\,s^{-1}$, respectively. These mobility values roughly correspond to an electron relaxation time, τ, between 100-130 fs at the Fermi energy of 120 meV[4]. The charge neutrality point (CNP) was identified at -0.069 V, indicating very low intrinsic doping.

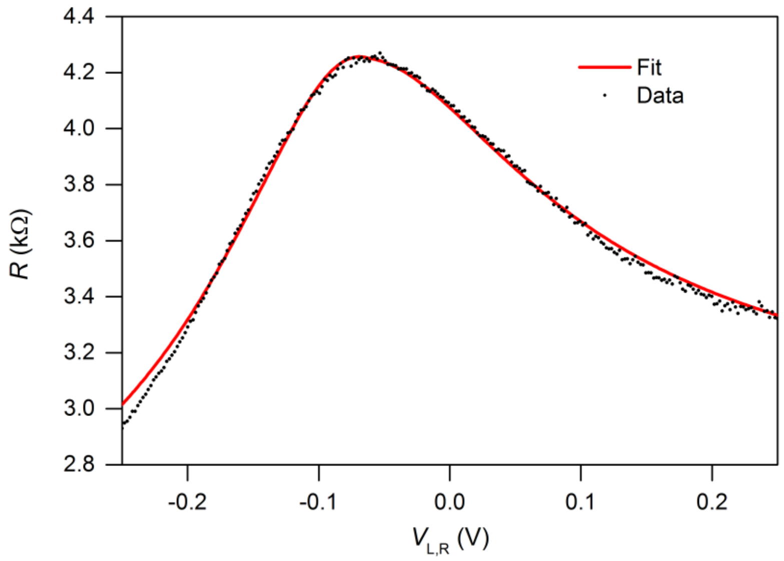

Supplementary Figure 2. **Electrical characterization of the graphene split-gate detector after brooming.** The diagram shows the two-terminal resistance between the source and drain contacts of the detector as a function of the gate voltage that was simultaneously applied to the right and left gates (black dots). The red curve shows the fit using the electrical circuit model from references [2,3]. Source data are provided as a Source Data file.

Suppl. Note 3. Schematic of the Fourier transform infrared spectroscopy setup

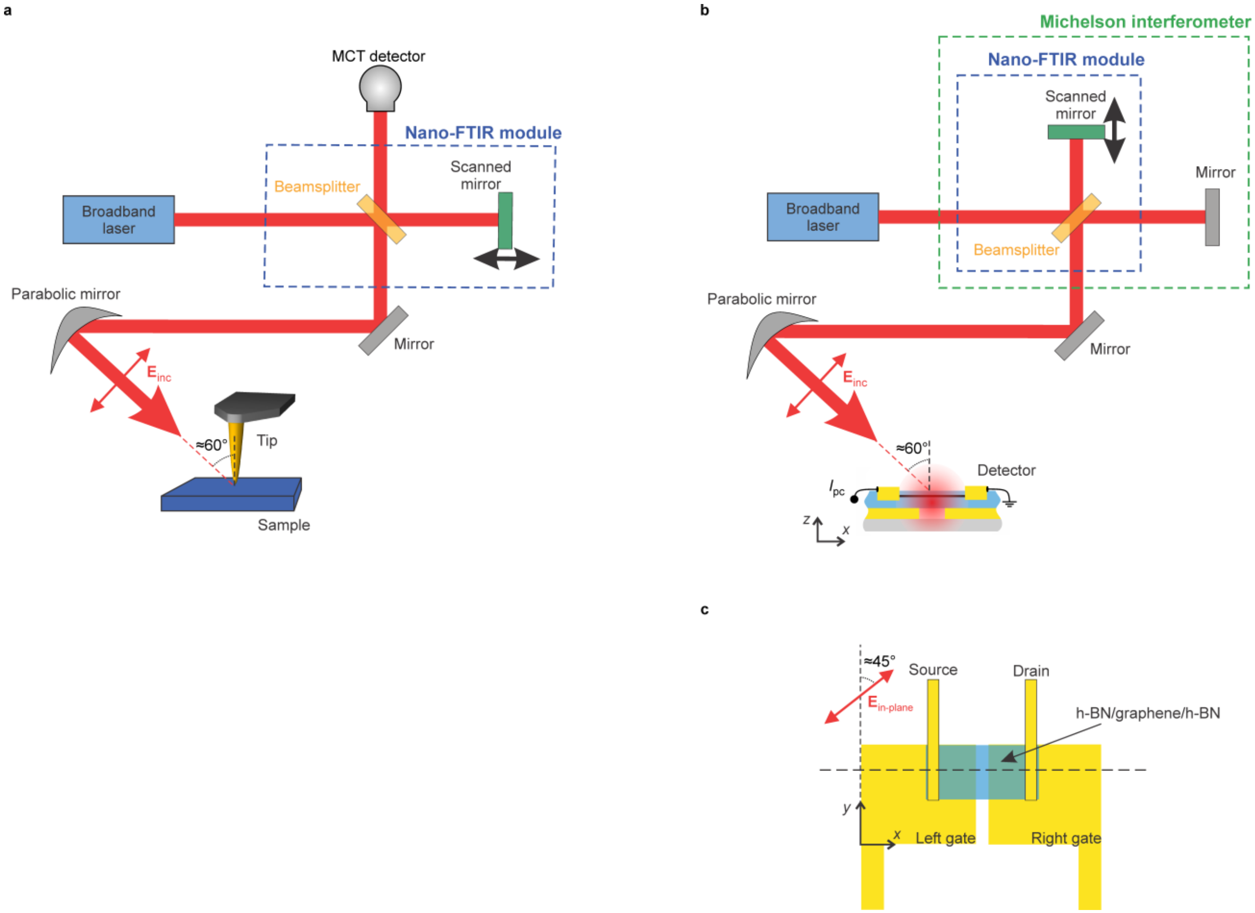

Supplementary Figure 3. Fourier transform infrared spectroscopy setup. a, Schematic of a standard scattering-type scanning near-field optical microscope (Neaspec/Attocube, Germany) comprising a nano-FTIR module marked by the dashed blue square. The nano-FTIR module consists of a ZnSe beamsplitter and a scanned mirror on a piezo-electric scanner. **b,** Schematic of the spectroscopy setup used in this work and described in the Methods section. The dashed blue square marks the nano-FTIR module. The dashed green square marks the Michelson interferometer. The angle of the incident radiation relative to the detector surface is about 30°. **c,** Schematic top view of the graphene split-gate detector. The red arrow indicates the projection of the incident electric field onto the detector surface.

Suppl. Note 4. Optical characterization of the graphene split-gate detector

To find the gate voltages where the photodetector yields the maximum photocurrent (PC), we measured I_{PC} as a function of the voltage on the left gate, V_L, and right gate, V_R (Suppl. Fig. 4a) using the spectroscopy setup described in Methods and Suppl. Note 3. The position of the scanning mirror of the interferometer was fixed during this measurement, yielding a spectrally integrated photocurrent. We found I_{max} = 11 nA for V_L = 0.15 V and V_R = −0.4 V (indicated by a white star in Suppl. Fig. 4a), where a p-n junction in graphene is created at the position of the gap between the two gates. We used these gate voltages to measure the spectra shown in Fig. 3b,f of the main text and Suppl. Fig. 5b, Suppl. Fig. 10a,b.

In Suppl. Fig. 4a we observe that the photocurrent exhibits several sign changes when the gate voltages were swept from negative to positive values. These sign changes are attributed to variations in both the type and concentration of the charge carriers in graphene above the left and right gates[3,5,6]. These sign changes allow us to estimate the charge neutrality point (CNP) voltages for the left and right gates, V_L^{CNP} = -0.15 V and V_R^{CNP} = -0.12 V, respectively, which we used in the electrostatic simulations (Suppl. Note 5.1). To better visualise the sign change pattern observed in Suppl. Fig. 4a, we provide a schematic illustration in Suppl. Fig. 4c. We recognise a 6-fold sign change pattern with the exception of a missing one sign change in the bottom left corner. The observation of the 6-fold pattern indicates that the photocurrent is based on the photo-thermoelectric effect, as demonstrated by other studies for split-gate graphene detectors in the mid-IR spectral range[3,5]. We speculate that the absence of a sign change in the bottom left corner was due to the limited ranges of the applied gate voltages and photocurrent contributions from graphene outside the split-gate (see Suppl. Fig. 1d).

For completeness, we show the non-normalized PC spectrum of the bare split-gate graphene detector and the emission spectrum of the broadband laser in Suppl. Fig. 5a and 5b, respectively. The non-normalized PC spectrum was measured at gate voltages at which the maximum spectrally integrated PC was obtained (V_L = 0.15 V, V_R = −0.4 V). To measure the emission spectrum of the broadband laser, we replaced the graphene split-gate detector in the spectroscopy setup with a conventional infrared nitrogen-cooled MCT detector (HgCdTe, mercury cadmium telluride, InfraRed Associates, USA). The MCT detector exhibits flat responsivity in the frequency range from 1100 to 1900 cm^{-1} and thus allows us to measure the emission spectrum of the laser.

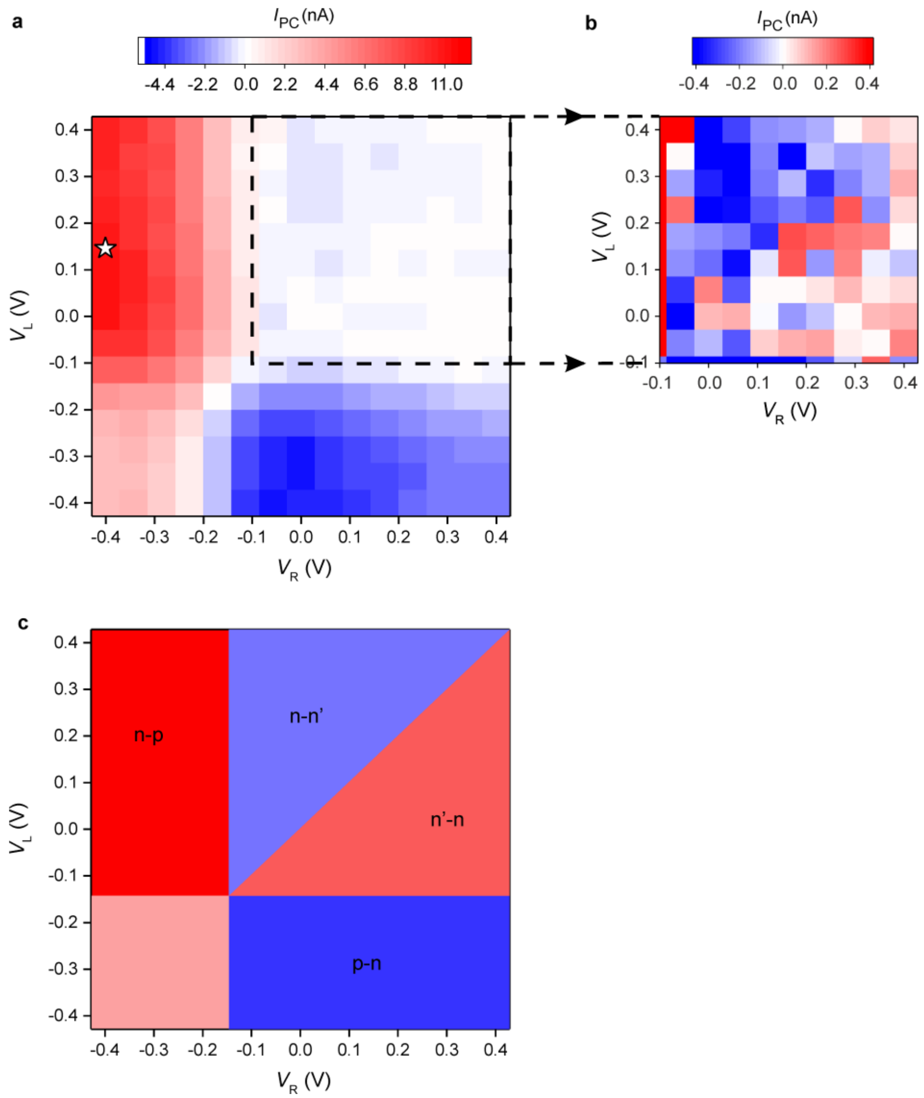

Supplementary Figure 4. a, Spectrally integrated photocurrent of the graphene split-gate detector as a function of voltage on the left and right gates under illumination of the broadband mid-IR laser. Black dashed square indicates the part of the colour plot shown in panel **b**. White star marks the gate voltages where the photocurrent is maximum. These gate voltages were used to measure the PC spectra in Fig. 3b,f of the main text and Suppl. Fig. 5b, Suppl. Fig. 13 of the Suppl. Note 4, 9. **b,** Area marked in panel **a** by black dashed square, but with enhanced contrast. **c,** Schematic of the PC as a function of the gate voltages shown in panel **a**. Source data are provided as a Source Data file.

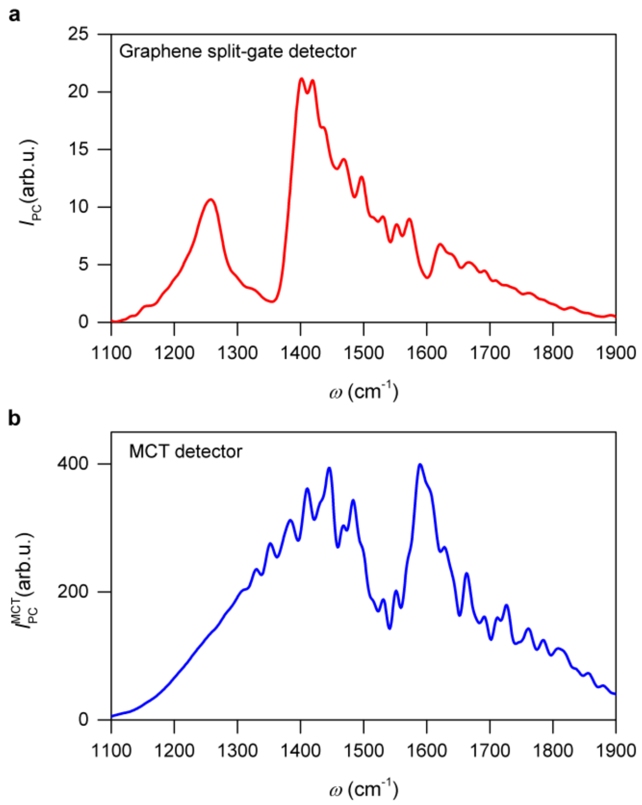

Supplementary Figure 5. a, Non-normalized PC spectra of the bare graphene split-gate detector for the gate voltages V_L = 0.15 V, V_R = -0.4 V, at which the maximum spectrally integrated PC was observed (see Suppl. Fig. 4a). **b,** Emission spectrum of the broadband mid-IR laser. The spectrum was measured using the same spectroscopy setup described in Methods and Suppl. Note 3 but with a conventional nitrogen-cooled infrared MCT detector (HgCdTe, mercury cadmium telluride, InfraRed Associates, USA), which has a spectrally flat responsivity in the frequency range from 1100 to 1900 cm^{-1}. Source data are provided as a Source Data file.

Suppl. Note 5. Calculation of optical absorption in graphene

To calculate the optical absorption in graphene (absorption in graphene), we used a two-dimensional (2D) detector geometry, that is, an infinite size of detector in the *y*-direction, which is justified by the device's elongated design along the y-axis (Suppl. Fig. 1a). First, we conducted an electrostatic simulation (see Suppl. Note 5.1) to determine the carrier concentration, ρ, in the graphene layer. Utilizing this carrier concentration, we calculated the chemical potential, μ, also known as the Fermi energy (see Suppl. Note 5.1, Suppl. Eq. 1). This allowed us to derive the optical conductivity of graphene, σ, as outlined in Suppl. Note 5.2 (Suppl. Eq. 3), from which we calculated the absorption in graphene through numerical electromagnetic simulation (see Suppl. Note 5.3), where the graphene was modelled as a current sheet (surface).

It is important to note that all calculations in the main text were performed assuming the incident light polarization is perpendicular to the detector's split-gate. To study the influence of polarization on graphene absorption, we also performed simulations with incident light polarized parallel to the split-gate (see Suppl. Fig. 6 in Suppl. Note 5.4).

Suppl. Note. 5.1. Electrostatic simulations

Electrostatic simulations were performed with the Electrostatics Module of the Comsol Multiphysics software. The electric potential was calculated by solving the Poisson equations after applying the appropriate voltages $\Delta V_{\mathrm{L}} = V_{\mathrm{L}} - V_{\mathrm{L}}^{\mathrm{CNP}} = 0.3$ V and $\Delta V_{\mathrm{R}} = V_{\mathrm{R}} - V_{\mathrm{R}}^{\mathrm{CNP}} = -0.28$ V to the left and right bottom split gates, respectively. The graphene channel was introduced as a grounded surface. The surface charge density in the graphene channel, $n(x)$, as a function of *x* coordinate was determined by Gauss´s law (Fig. 2b of the main text). The static dielectric constants of Si, SiO_2 and h-BN were set to 11.69[7], 3.1[8] and 4.3[9], respectively.

The graphene chemical potential, μ, was obtained from [5]:

$$n(x) = \frac{2e}{\pi(\hbar v_{\mathrm{F}})^2}\int_0^\infty \epsilon\big[f\big(\epsilon,-\mu(x)\big) + f\big(\epsilon,\mu(x)\big)\big]\,\mathrm{d}\epsilon, \quad (1)$$

where ϵ is electron energy, e is electron charge, ħ is reduced Planck's constant, $f\big(\epsilon,\mu(x)\big) = [e^{(\epsilon-\mu(x))/T} + 1]^{-1}$ is the Fermi-Dirac distribution, $v_{\mathrm{F}} = 10^6$ m s^{-1} is Fermi velocity, T is the temperature in the energy units.

Suppl. Note. 5.2. Dielectric functions of materials used in electromagnetic simulations

The dielectric infrared permittivity tensor of h-BN was modelled according to the following equation:

$$\varepsilon_{\mathrm{h-BN}}^{j}(\omega) = \varepsilon_{\infty}^{j}\left[1 + \frac{\left(\omega_{\mathrm{LO}}^{j}\right)^2 - \left(\omega_{\mathrm{TO}}^{j}\right)^2}{\left(\omega_{\mathrm{TO}}^{j}\right)^2 - \omega^2 - \mathrm{i}\omega\Gamma^{j}}\right], \quad (2)$$

where, $j = \parallel, \perp$ indicates the parallel and perpendicular directions to the optical axis, ω_{TO}^{j} and ω_{LO}^{j} are the transverse optical (TO) and longitudinal optical (LO) phonon frequencies, Γ^{j} is the damping constant, and ε_{∞}^{j} is the high-frequency permittivity. We took $\omega_{\mathrm{TO}}^{\parallel}$ = 760 cm^{-1}, $\omega_{\mathrm{LO}}^{\parallel}$ = 825 cm^{-1}, $\omega_{\mathrm{TO}}^{\perp}$ = 1362.7 cm^{-1}, $\omega_{\mathrm{LO}}^{\perp}$ = 1616.9 cm^{-1}, $\varepsilon_{\infty}^{\parallel}$ = 2.95, $\varepsilon_{\infty}^{\perp}$ = 4.98, $\Gamma^{\parallel}$ = 3 cm^{-1}, $\Gamma^{\perp}$ = 7.3 cm^{-1} according to ref [10].

The two-dimensional optical conductivity of graphene, $\sigma(\omega, x)$, was calculated using the Kubo formula[11] according to the following formulas:

$$\sigma(\omega, x) = \sigma_{\mathrm{intra}}(\omega, x) + \sigma_{\mathrm{inter}}(\omega, x), \quad (3)$$

$$\sigma_{\mathrm{intra}}(\omega, x) = \frac{2\mathrm{i}e^2T}{\pi\hbar\Omega}\ln\left[2\cosh\left(\frac{\mu(x)}{2T}\right)\right], \quad (4)$$

$$\sigma_{\mathrm{inter}}(\omega, x) = \frac{\mathrm{i}e^2\Omega}{\pi\hbar^2}\int_0^{\infty}\frac{f(-\epsilon, \mu(x)) - f(\epsilon, \mu(x))}{\Omega^2 - 4(\epsilon/\hbar)^2}\mathrm{d}\epsilon, \quad (5)$$

where Ω was defined as $\Omega = \omega + \mathrm{i}\tau^{-1}$, τ = 200 fs is the assumed electron relaxation time, μ is graphene chemical potential (Fermi energy), $f(\epsilon, \mu(x)) = [e^{(\epsilon-\mu(x))/T} + 1]^{-1}$ is the Fermi-Dirac distribution and $\partial_\epsilon = \partial/\partial\epsilon$.

The permittivity of CBP was modelled using the Drude−Lorentz model assuming 3 oscillators[12]:

$$\varepsilon_{\mathrm{CBP}}(\omega) = \varepsilon_{\infty} + \sum_k \frac{{S_k}^2}{\omega_k^2 - \omega^2 - \mathrm{i}\omega\gamma_k}, k = 1 - 3, \quad (6)$$

where S_k, ω_k and γ_k represent the intensity, central frequency and damping of the *k*-th oscillator. For the dielectric non-dispersive background, ε_∞, we used the value ε_∞= 2.8. The two main molecular vibrations in the considered range are at ω_1 = 1450 cm^{-1} (γ_1 = 8.3, S_1 = 158 cm^{-1}) and ω_2 = 1504 cm^{-1} (γ_2 = 13.4 cm^{-1}, S_2 = 164 cm^{-1}). The third weaker vibration is characterized by ω_3 = 1478 cm^{-1} (γ_3 = 3 cm^{-1}, S_3 = 59 cm^{-1}).

The dielectric function of gold was modelled by Drude model $\varepsilon_{\mathrm{Au}}(\omega) = 1 - \omega_{\mathrm{p}}^2/(\omega^2 - \mathrm{i}\Gamma\omega)$ with the plasma frequency ω_{p} = 73494.85 cm^{-1} and the plasma collision rate Γ = 567.8 cm^{-1}. The parameters were obtained from fitting dielectric data from ref[13].

The dielectric function of SiO_2 was modelled by Drude–Lorentz model assuming 3 oscillators:

$$\varepsilon_{\mathrm{SiO2}}(\omega) = \varepsilon_{\mathrm{SiO2},\infty} + \sum\nolimits_i \frac{s_i\omega_i^2}{\omega_i^2 - \omega^2 - \mathrm{i}\omega\gamma_i}, i = 1 - 3, \quad (7)$$

where s_i, ω_i and γ_i represent the strength, central frequency and damping of the i-th oscillator. The parameters were obtained from fitting dielectric data from ref[14] for Silicon dioxide (SiO2) glass. For the high-frequency permittivity, $\varepsilon_{\mathrm{SiO2},\infty}$, we assumed the value $\varepsilon_{\mathrm{SiO2},\infty}$= 2.1 since it provided a good fit. All fit parameters are shown in Suppl. Table 1.

i	ω_i (cm^{-1})	γ_i, (cm^{-1})	s_i
1	1072.27	67.2179	0.6752
2	805.20	75.7996	0.0929
3	457.61	44.5775	1.0218

Suppl. Table 1. Parameters for the dielectric function of SiO_2.

For the dielectric function of Si substrate we used $\varepsilon_{\mathrm{Si}} = 11.69$.

Suppl. Note. 5.3. Electromagnetic simulations

Electromagnetic simulations were performed with the radio frequency (RF) module of the COMSOL Multiphysics software. We employed the scattered field formulation, wherein the background field—represented by a plane wave in our study—was analytically defined. In the simulations, the graphene is defined as a current sheet (surface) within the boundary conditions, $\mathbf{j}(x) = \sigma(x)\mathbf{E}$, where σ is the two-dimensional optical graphene conductivity (Suppl. Eq. 3). The absorption in graphene, α, is obtained according to:

$$\alpha = \frac{1}{2}\int_{-D/2}^{D/2} \mathrm{Re}(\mathbf{j}(x)\cdot\mathbf{E}^*)\ \mathrm{d}x, \quad (8)$$

where the symbols $*$ indicated conjugation and D is the width of the graphene channel.

The size of the computational domain was 48×48 μm^2 with scattering boundary conditions employed on the lateral boundaries. We used an inhomogeneous free-triangular mesh, where the minimal element size was 0.3 nm in the graphene channel, increasing up to 1 μm at the boundaries of the computational domain.

Suppl. Note. 5.4. Influence of incident light polarization on absorption in graphene

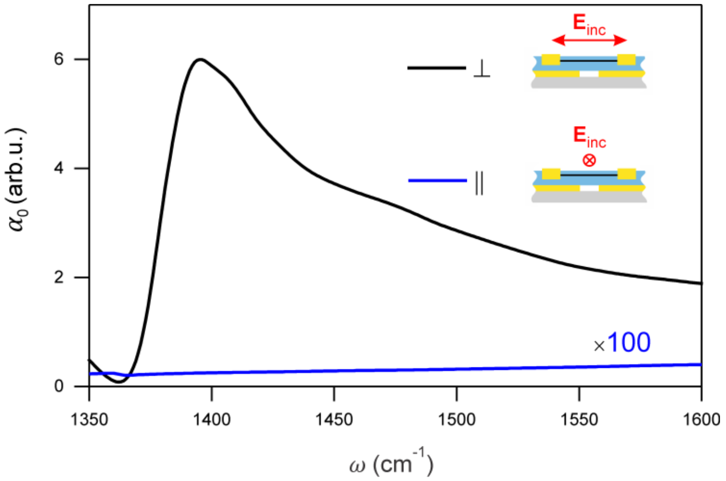

Supplementary Figure 6. Influence of incident light polarization on the absorption in graphene. Black and blue curves show the simulated absorption in graphene when the incident light polarization is perpendicular and parallel to the split gate, respectively. The values of the blue curve were multiplied by 100 for visual comparison. The top and bottom inset images illustrate incident light polarization perpendicular and parallel to the split gate, respectively. Source data are provided as a Source Data file.

Suppl. Note 6. Influence of gap size, h-BN thickness and HPhPs on graphene absorption

To study the influence of the gap size of the split gate, L_{gap}, and the h-BN thicknesses, $t_{\mathrm{top,bottom}}$, (indicated in Suppl. Fig. 7a) on the performance on the detector and the molecular vibrational contrast Δ (defined in Fig. 3 of the main text and in Suppl. Fig. 8,9), we performed numerical simulations of the absorption in graphene, $\alpha_{0,\mathrm{CBP}}(\omega)$, similar to those in Fig. 2c,d and Fig. 3c of the main text, but with varying L_{gap} and $t_{\mathrm{top,bottom}}$. Additionally, to study and highlight the enhancement of Δ due to the HPhPs in h-BN, we compare the results with simulations performed in absence of HPhPs in h-BN, where we replaced the frequency-dependent permittivity tensor of h-BN (see Suppl. Note 5.2) by a frequency-independent permittivity tensor with $\varepsilon_{\mathrm{h-BN}}^{\perp}(\omega) = \varepsilon_{\infty}^{\perp} = 4.98$ and $\varepsilon_{\mathrm{h-BN}}^{\parallel}(\omega) = \varepsilon_{\infty}^{\parallel} = 2.95$.

We note that all simulations in the main text were performed for a detector with a split gate gap size of L_{gap} = 160 nm and top and bottom h-BN layer thicknesses of t_{top} = 3 nm and t_{bottom} = 5 nm, respectively, to which we refer to as the main text parameters in the following discussion.

We find that in the presence of HPhPs, the absorption in graphene increases, exhibiting a peak that shifts to higher frequencies with increasing h-BN thickness (Fig. 7b) and decreasing gap width (Fig. 7c). Both the increased absorption and peak shifts are typical characteristics of localized resonant HPhP modes in h-BN nanostructures[12,15]. In the detector, these resonances emerge due to the reflection of the HPhPs in the h-BN above the gap region at the Au edges, owing to the large momentum mismatch between HPhPs in the h-BN above the gap region and the metallic split gate[16]. These results highlight the role of HPhPs in absorption in graphene, demonstrating the potential for tuning and increasing absorption by varying detector parameters such as h-BN thickness and gap width.

When a 10 nm layer of molecules is placed directly on top of the detector (Suppl. Fig. 7c,d), all spectra exhibit dips at the molecular vibration frequencies (marked by the three vertical dashed blue lines). The depths of these molecular-vibrational resonance dips are larger when HPhPs are present in the h-BN layers, as discussed in the main text. Importantly, we do not observe pronounced differences in dip depth for detectors with varying gap sizes and h-BN thicknesses. This indicates that the enhanced depth of these molecular-vibrational resonance dips is primarily caused by the interaction of molecular vibrations with non-resonant HPhP modes, rather than with the resonant HPhPs mode, which depends on the structural parameters of the detector. Larger enhancements of the molecular resonance dip depths are expected when the resonant HPhP mode overlaps with molecular vibrational resonances.

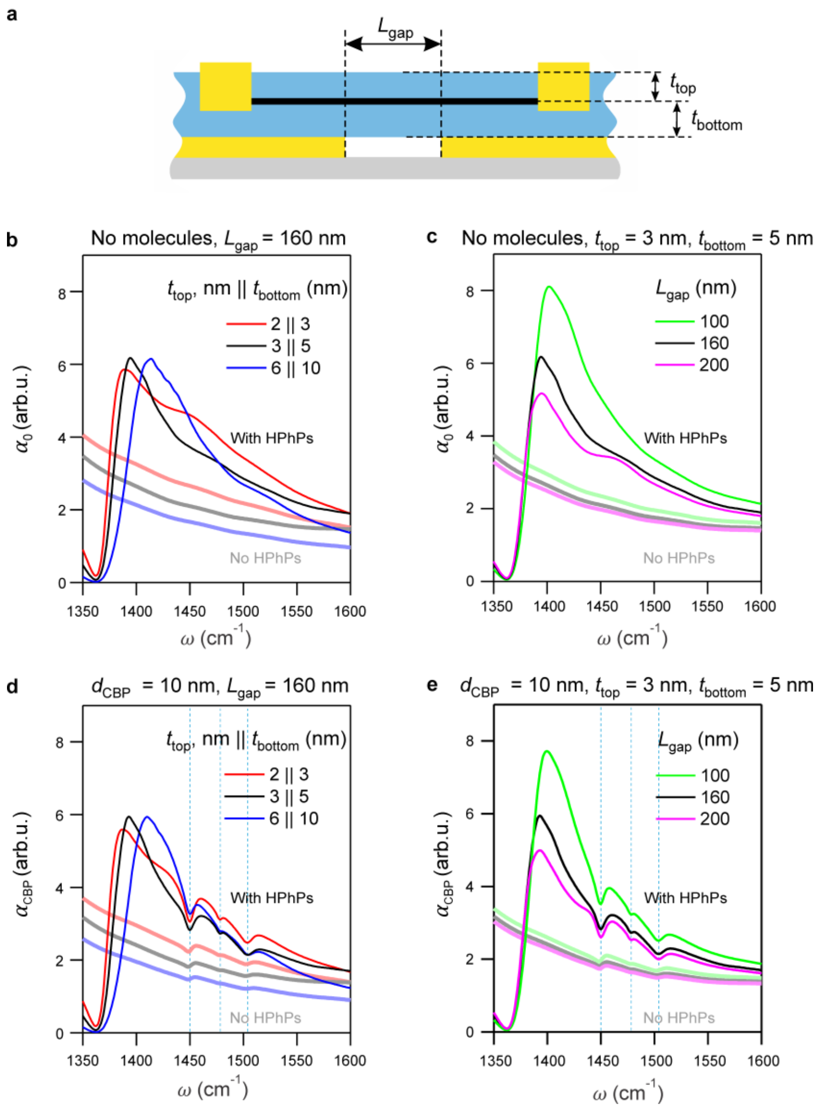

Supplementary Figure 7. **Absorption in graphene in detectors with different h-BN thicknesses and gap sizes. a,** Schematic of the detector with the gap size of the split gate, L_{gap}, and top and bottom h-BN layers with thicknesses t_{top} and t_{bottom}, respectively. **b**, Absorption in graphene in the bare detector, α_0, with the fixed L_{gap} = 160 nm but different $t_{top}$and t_{bottom}. The red, black and blue curves show α_0 in the presence HPhPs and the light red, grey, light blue curves show α_0 in the absence of the HPhPs. **c**, Absorption in graphene in the bare detector, α_0, with fixed t_{top} = 3 nm, t_{bottom} = 5 nm but different L_{gap}. The magenta, black and green curves show α_0 in the presence HPhPs and the light magenta, grey, light green curves show α_0 in the absence of the HPhPs. **d,e** Same as **b,c** but with 10 nm-thick CBP molecules on top of the detector. The three vertical dashed blue lines indicate the frequencies of the molecular vibrational resonances of CBP. Source data are provided as a Source Data file.

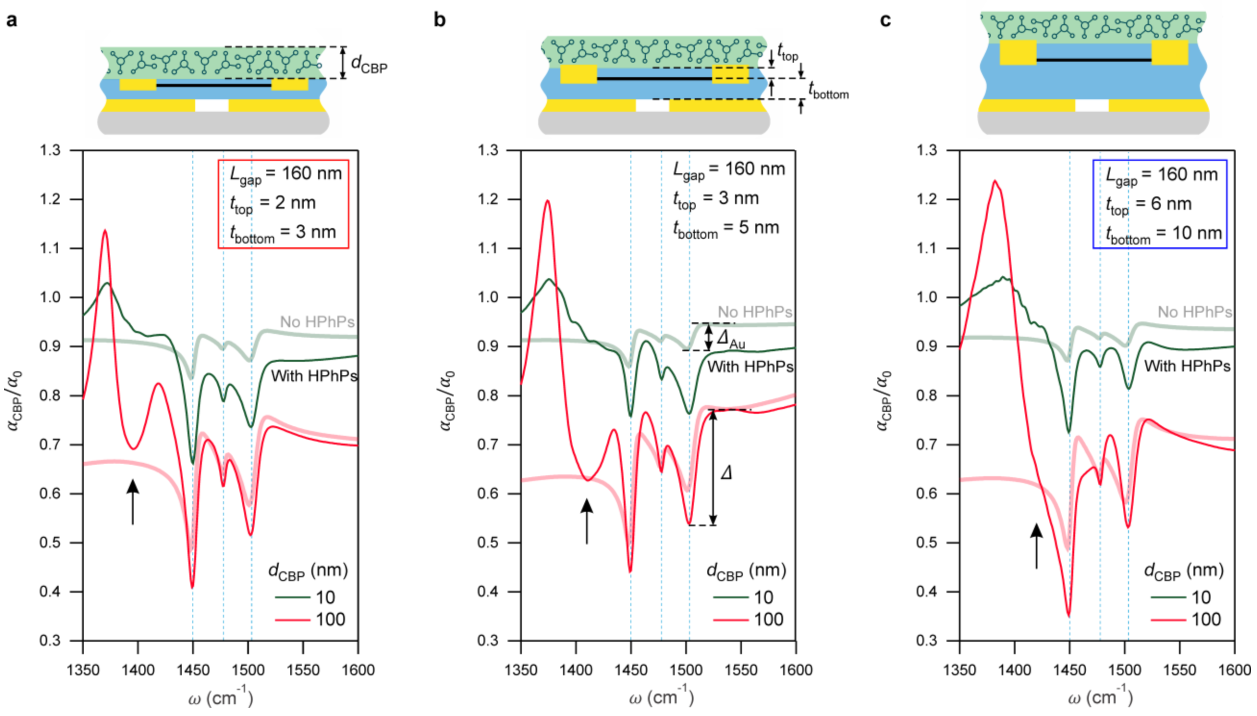

Supplementary Figure 8. **Normalized absorption in graphene in detectors with different thicknesses of h-BN layers. a,** Normalized absorption in graphene, $\alpha_{\text{CBP}}/\alpha_0$, for different thicknesses of CBP layers placed directly on top of the detector with the gap size L_{gap} = 160 nm and thicknesses of the h-BN layers t_{top} = 2 nm, t_{bottom} = 3 nm. The red and green curves show $\alpha_{\text{CBP}}/\alpha_0$ when there are HPhPs in h-BN. The light red and light green curves show $\alpha_{\text{CBP}}/\alpha_0$ when there are no HPhPs in h-BN. **b,c,** The same as in panel **a** but for the t_{top} = 3 nm, t_{bottom} = 5 nm and t_{top} = 6 nm, t_{bottom} = 10 nm, respectively. **b**, Black arrows indicate the molecular-vibrational contrasts, Δ and Δ_{Au}, when there are HPhPs and no HPhPs in the h-BN. **a,b,c,** Three vertical dashed blue lines indicate the frequencies of the molecular vibrational resonances. Source data are provided as a Source Data file.

In Suppl. Fig. 8 and 9, we show the normalized absorption spectra, $\alpha_{\text{CBP}}(\omega)/\alpha_0(\omega)$, for 10 and 100 nm thick molecular layers on top of the detector in the presence and absence of HPhPs in the h-BN. Importantly, the molecular vibrational contrast, Δ remains almost constant for detectors with different structural parameters, which is consistent with behaviour of molecular-vibrational resonance dips in Suppl. Fig. 7 (discussed in the previous paragraph). Interestingly, in the presence of HPhPs in h-BN we observe that spectra exhibit different baselines. This difference is particularly evident in the spectra for a 100 nm thick molecular layer, where a frequency of a dip (marked by a black arrow) shifts to higher frequencies with increasing h-BN thicknesses (Suppl. Fig. 8) and with decreasing gap size. These changes in the baseline of the normalized spectra are attributed to the shift in the maximum of non-normalized absorption in graphene in Suppl. Fig. 7b,c.

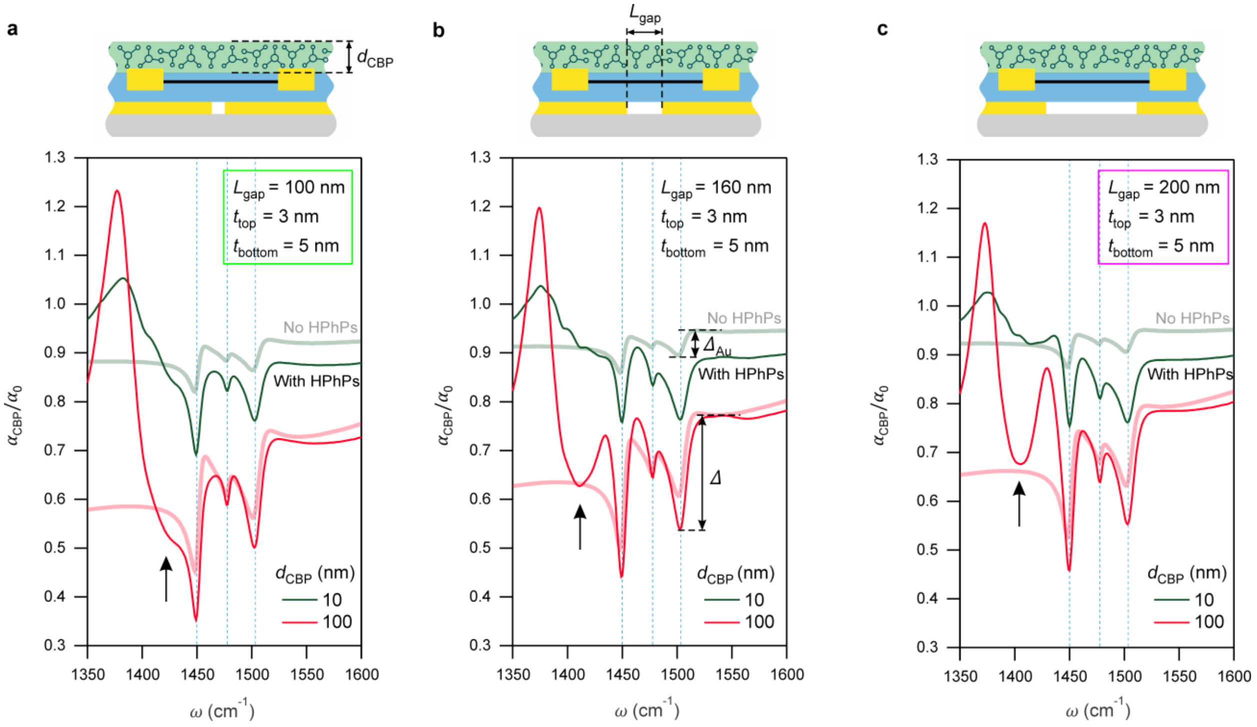

Supplementary Figure 9. Normalized absorption in graphene in detectors with different gap sizes. a, Normalized absorption in graphene, $\alpha_{\mathrm{CBP}}/\alpha_0$, for different thicknesses of CBP layers placed directly on top of the detector with the gap size L_{gap} = 100 nm and thicknesses of the h-BN layers t_{top} = 3 nm, t_{bottom} = 5 nm. The red and green curves show $\alpha_{\mathrm{CBP}}/\alpha_0$ when there are HPhPs in h-BN. The light red and light green curves show $\alpha_{\mathrm{CBP}}/\alpha_0$ when there are no HPhPs in h-BN. **b,c,** The same as in panel **a** but for the L_{gap} = 160 nm and 200 nm, respectively. **b**, Black arrows indicate the molecular-vibrational contrasts, Δ and Δ_{Au}, when there are HPhPs and no HPhPs in the h-BN. **a,b,c,** Three vertical dashed blue lines indicate the frequencies of the molecular vibrational resonances. Source data are provided as a Source Data file.

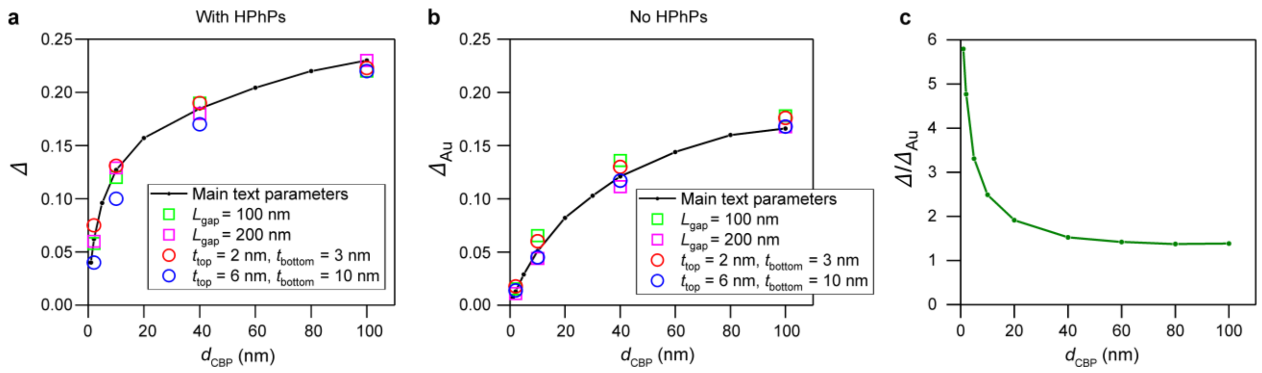

Supplementary Figure 10. Dependence of the molecular-vibrational contrast, Δ, at 1508 cm^{-1}. a, The black curve (values from the red curve in Fig. 4a of the main text) shows molecular-vibrational contrast, Δ, (indicated in Suppl. Fig. 8 and Suppl. Fig. 9) extracted from the simulated spectra of normalized absorption in graphene, $\alpha_{\mathrm{CBP}}/\alpha_0$, when there are HPhPs in h-BN. The symbols show Δ for the detectors with different gap sizes and thicknesses of h-BN layers. **b,** the same as in panel **a** but in absence of HPhPs in h-BN. **c,** The green curve shows the enhancement of molecular-vibrational contrast, $\Delta/\Delta_{\mathrm{Au}}$, due to the presence of HPhPs calculated from simulated values from the panel **a** and **b**. Source data are provided as a Source Data file.

To quantify the influence of the gap size of the split gate and h-BN thickness on the molecular-vibrational contrast, we measured the depth of this contrast at 1508 cm^{-1} both in the presence and absence of HPhPs in h-BN (denoted Δ and Δ_{Au}, respectively, and illustrated in Suppl. Fig. 8 and Suppl. Fig. 9) and plot them in Suppl. Fig. 10a,b as a function of the layer thickness d_{CBP}. As discussed in the previous paragraph, we find that the Δ-values are almost independent of the detector's structural parameters due to the frequency mismatch between the HPhP resonance and the molecular vibrational resonances. To quantify the enhancement of the molecular-vibrational contrast due to merely HPhPs, we plot the ratio $\Delta/\Delta_{\mathrm{Au}}$ in Suppl. Fig. 10c. For the 10 nm molecular layer, we found that this ratio $\Delta/\Delta_{\mathrm{Au}}$ is approximately 2.5. We explain this enhancement by the additional field concentration near the detector surface provided by HPhPs. Source data are provided as a Source Data file.

Suppl. Note 7. Influence of CBP background permittivity on normalized absorption in graphene in the molecule-covered detector

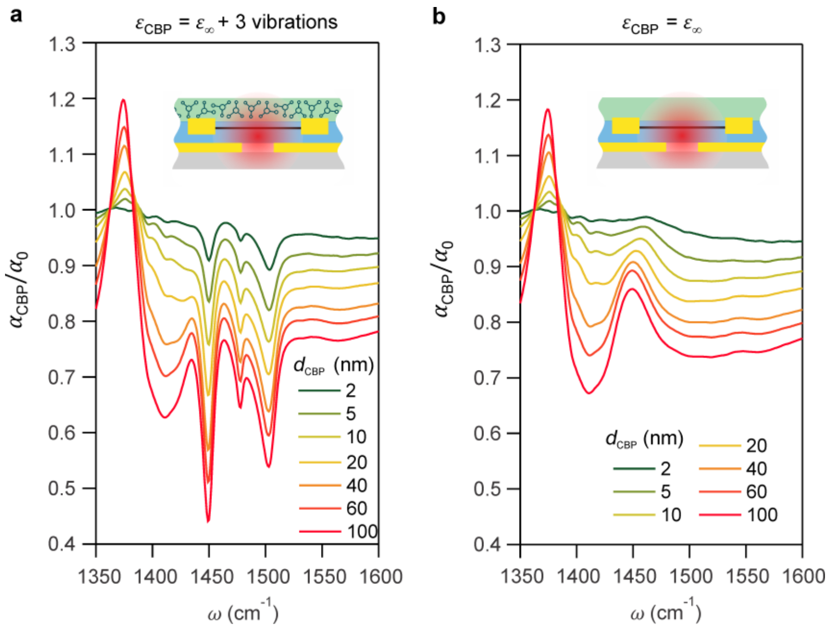

Supplementary Figure 11. **Simulated normalized absorption in graphene for differently thick CBP layers placed directly on top of the detector. a,** The full dielectric function of CBP molecules, $\varepsilon_{\mathrm{CBP}}(\omega)$, was considered in the simulations. **b,** Only the background permittivity of CBP molecules was considered in the simulations, $\varepsilon_{\mathrm{CBP}}(\omega) = \varepsilon_\infty = 2.8$. Source data are provided as a Source Data file.

Suppl. Note 8. Convolution of the normalized absorption and transmission spectra

To compare our experimental data with theoretical simulations, we need to ensure that both have the same spectral resolution. Our experimental spectra shown in Fig. 3b,f of the main text have a spectral resolution of about 6.5 cm^{-1}. The experimental spectra were obtained by applying a Fourier Transform (FT) to the interferogram using a Hann window and zero padding. To match this spectral resolution in our simulations, we first perform an FT on the zero-padded Hann window. This gives us a function that captures the spectral characteristics of our measurement setup. We then convolve this function with the simulation data. Suppl. Fig. 12 shows the simulations spectra before and after convolution. This convolution ensures that the simulation data has the same spectral resolution as the experimental data, allowing for a direct and accurate comparison.

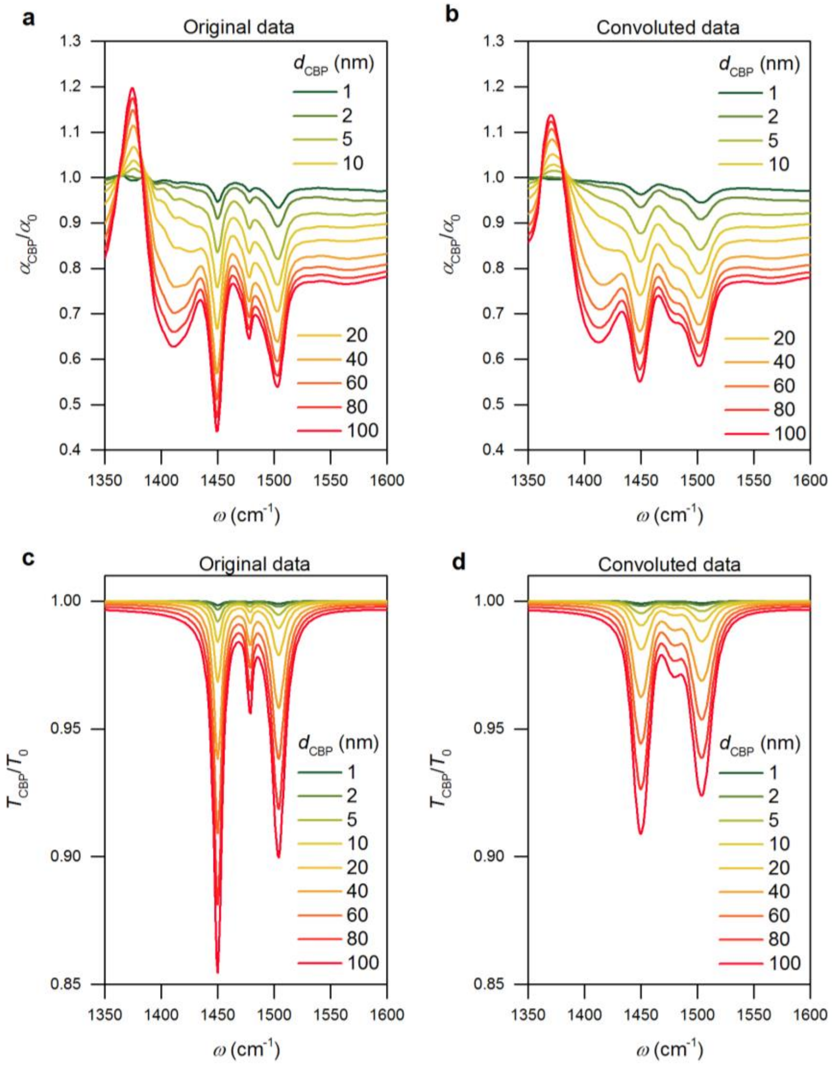

Supplementary Figure 12. Comparison of convoluted and original simulated spectra. a,b, Original and convoluted simulated normalized absorption in graphene for different thicknesses of CBP layers placed directly on top of the detector, respectively. All spectra are normalized to the absorption in graphene without molecules on top of the detector. **c,d,** Original and convoluted **s**imulated normalized transmission spectra of differently thick CBP layers on a CaF_2 substrate, respectively. Source data are provided as a Source Data file.

Suppl. Note 9. Data set of the experimental on-chip SEIRA and far-field transmission spectra

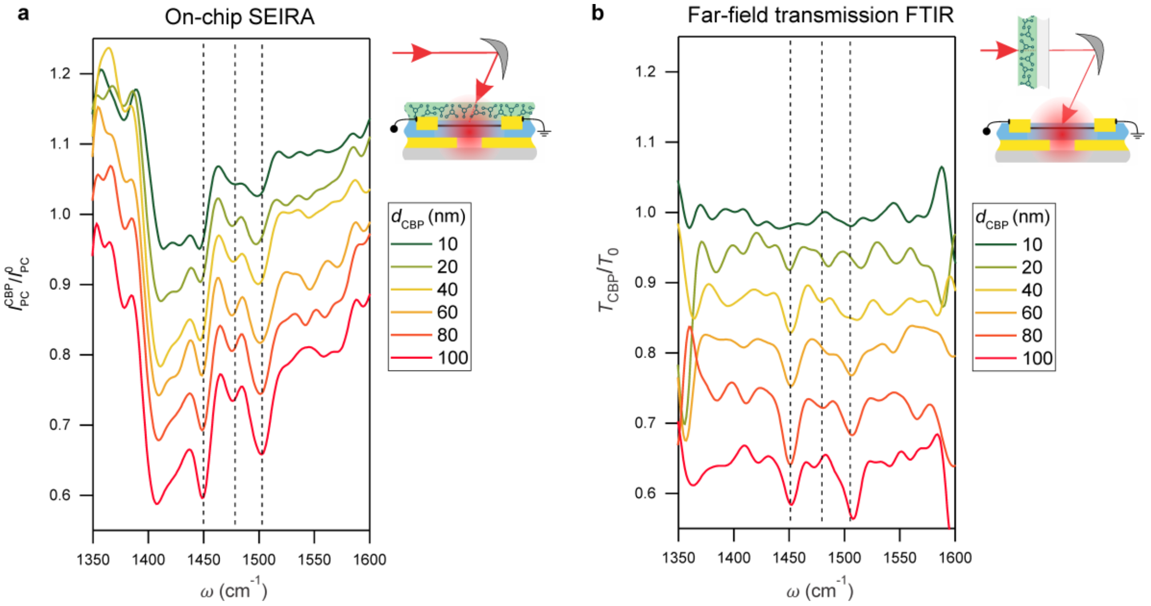

Supplementary Figure 13. Full data set of the experimental spectra. a, Experimental PC spectra for differently thick CBP layers deposited on top of the detector. All spectra are normalized to the PC spectrum of the bare detector. **b**, Experimental PC spectra of the bare detector when differently thick CBP layers on a CaF_2 substrate are placed in front of the detection at centimetre distance. A linear baseline was subtracted from all spectra. All spectra are normalized to the PC spectrum obtained with a bare CaF_2 substrate. **a,b,** Spectra are offset along the *y*-axis for clarity. Source data are provided as a Source Data file.

Supplementary references

1. Lindvall, N., Kalabukhov, A. & Yurgens, A. Cleaning graphene using atomic force microscope. *J. Appl. Phys.* **111**, 064904 (2012).
2. Wang, L. *et al.* One-dimensional electrical contact to a two-dimensional material. *Science* **342**, 614–617 (2013).
3. Castilla, S. *et al.* Plasmonic antenna coupling to hyperbolic phonon-polaritons for sensitive and fast mid-infrared photodetection with graphene. *Nat. Commun.* **11**, 4872 (2020).
4. Zhu, W., Perebeinos, V., Freitag, M. & Avouris, P. Carrier scattering, mobilities, and electrostatic potential in monolayer, bilayer, and trilayer graphene. **80**, 235402 (2009).
5. Woessner, A. *et al.* Electrical detection of hyperbolic phonon-polaritons in heterostructures of graphene and boron nitride. *npj 2D Mater. Appl.* **1**, 25 (2017).
6. Lemme, M. C. *et al.* Gate-Activated Photoresponse in a Graphene p-n Junction. *Nano Lett.* **11**, 4134–4137 (2011).
7. Kittel, C. *Introduction to solid state physics*. (John Wiley & Sons, Inc, 1957).
8. Joshi, B. & Mahajan, A. Growth and characterization of porous SiO2 thin films for interlayer dielectrics applications in ULSI. *Optoelectron. Adv. Mater.* **1**, 659–662 (2007).
9. Jang, S. K., Youn, J., Song, Y. J. & Lee, S. Synthesis and Characterization of Hexagonal Boron Nitride as a Gate Dielectric. *Sci. Rep.* **6**, 8583–8590 (2016).
10. Giles, A. J. *et al.* Ultralow-loss polaritons in isotopically pure boron nitride. *Nat. Mater.* **17**, 134–139 (2018).
11. Falkovsky, L. A. Optical properties of graphene and IV - VI semiconductors. *Uspekhi Fiz. Nauk* **51**, 887–897 (2008).
12. Autore, M. *et al.* Boron nitride nanoresonators for Phonon-Enhanced molecular vibrational spectroscopy at the strong coupling limit. *Light Sci. Appl.* **7**, 17172–17178 (2018).
13. Johnson, P. B. & Christy, R. W. Optical constants of the noble metals. *Phys. Rev. B* **6**, 4370 (1972).
14. Palik, E. D. *Handbook of Optical Constants of Solids*. (Academic Press, 1985).
15. Dolado, I. *et al.* Remote near-field spectroscopy of vibrational strong coupling between organic molecules and phononic nanoresonators. *Nat. Commun.* **13**, 6850 (2022).
16. Yuan, Z. *et al.* Extremely Confined Acoustic Phonon Polaritons in Monolayer-hBN/Metal Heterostructures for Strong Light-Matter Interactions. *ACS Photonics* **7**, 2610–2617 (2020).
